# Identifying Prostate Cancer Among Men with Lower Urinary Tract Symptoms

**DOI:** 10.1016/j.euros.2020.12.004

**Published:** 2021-01-01

**Authors:** Tobias Nordström, Jan Chandra Engel, Martin Bergman, Lars Egevad, Markus Aly, Martin Eklund, Thorgerdur Palsdottir, Henrik Grönberg

**Affiliations:** a Department of Clinical Sciences at Danderyd Hospital, Karolinska Institutet, Stockholm, Sweden; b Department of Medical Epidemiology and Biostatistics, Karolinska Institutet, Stockholm, Sweden; c Department of Oncology-Pathology, Karolinska Institutet, Stockholm, Sweden; d Department of Molecular Medicine and Surgery, Karolinska Institutet, Stockholm, Sweden

**Keywords:** Prostate cancer, Prostate neoplasm, Benign prostate hyperplasia, Biomarker, STHLM3, Stockholm3

## Abstract

**Background:**

In men aged above 50 yr, lower urinary tract symptoms (LUTS), benign prostate hyperplasia, and prostate cancer are common urological conditions. Current guidelines for general practitioners frequently recommend prostate-specific antigen (PSA) testing in patients with LUTS for the detection of prostate cancer.

**Objective:**

To assess the performance of PSA, PSA density, and the Stockholm3 blood test for identification of prostate cancer among men with LUTS.

**Design, setting, and participants:**

In this post hoc analysis of a population-based diagnostic trial (STHLM3, *n* = 58 588), 4588 men aged 50–69 yr, without previous prostate cancer, with International Prostate Symptom Score (IPSS) data, and having PSA ≥ 3 ng/mL were identified. Men with at least moderate LUTS, defined as an IPSS score of ≥8, were included. PSA density and Stockholm3 scores were calculated.

**Intervention:**

Participants underwent 10–12-core systematic prostate biopsies.

**Outcome measurements and statistical analysis:**

The primary outcome was significant prostate cancer (sPCa) defined as International Society of Urological Pathology (ISUP) grade ≥2. Logistic regression analysis adjusted for age and previous biopsy status was performed. The area under the receiver operating characteristic curve (AUC) was calculated, and decision curve analysis was performed.

**Results and limitations:**

Out of 4588 men, 1544 (34%) reported at least moderate LUTS. The median age was 64 yr, and 11% had undergone a previous prostate biopsy. The Stockholm3 test showed superior discrimination for sPCa to PSA density, which in turn showed superior discrimination to PSA (AUC 0.77 vs 0.70 vs 0.61, *p* <  0.02). Calibration of the Stockholm3 test was adequate. Performing biopsy only in men with PSA ≥5 ng/mL saved 64% of biopsies, but resulted in missing 52% of detectable sPCa. Recommending biopsy for men with PSA density ≥0.07 resulted in sparing 26% of biopsy procedures and delaying the diagnosis of 12% of sPCa cases, with a 6.1% risk of sPCa among unbiopsied men. Recommending men with Stockholm3 ≥ 0.11 for biopsy resulted in sparing 53% of biopsy procedures and delaying the diagnosis of 20% of sPCa cases, with a 5.1% risk of finding sPCa in unbiopsied men.

**Conclusions:**

PSA density and the Stockholm3 blood test were superior to PSA for the identification of prostate cancer among men with LUTS.

**Patient summary:**

In this analysis of a large Swedish study, we find that the use of prostate-specific antigen (PSA) density or the Stockholm3 blood test instead of only PSA might improve the detection of prostate cancer among men with lower urinary tract symptoms.

## Take Home Message

The use of the Stockholm3 blood test or prostate-specific antigen density might improve the detection of prostate cancer among men with lower urinary tract symptoms.

## 1 Introduction

More than a fourth of men aged above 50 yr exhibit at least moderate lower urinary tract symptoms (LUTS), and the risk of developing symptomatic benign prostatic hyperplasia (BPH) has been suggested to be 46% [1], [2]. Together with the fact that approximately 450 000 European men are diagnosed with prostate cancer yearly, this illustrates that LUTS, BPH, and prostate cancer are common urological conditions.

The relationship between prostate cancer, BPH, and LUTS has been studied in various settings [2], [3], [4], [5]. There are some indications that LUTS might lack significant associations with the risk of prostate cancer [6]. However, no consensus has yet been reached on the relationship between LUTS and the risk of cancer. LUTS have most often benign causes but prostate cancer might coexist [7], and guidelines frequently recommend testing with prostate-specific antigen (PSA) as a part of the diagnostic workup of men with urinary symptoms [8].

In line with this, it has previously been shown that two-thirds of general practitioners would recommend PSA testing in patients with LUTS [9]. In addition, studies show that men with LUTS expect to be tested for the presence of prostate cancer [10]. Studies from Australia, where the National Health and Medical Research Council advises against PSA testing on the basis of LUTS, have shown that 75% of patients with LUTS nonetheless expect to be tested for prostate cancer [11].

Whether to recommend early detection for prostate cancer or not is an ongoing debate. With few exceptions, currently no formal nationwide screening programs are implemented for prostate cancer. However, opportunistic screening is widespread [12]. It has been shown that only 1% of the general population is aware that prostate cancer can manifest without symptoms [10].

In the light of this, it is important that tests used for prostate cancer detection in men with LUTS are well characterized and that these men are offered well-defined test strategies of high quality. Despite this, the European Association of Urology and National Institute for Health and Care Excellence guidelines currently only suggest that PSA is considered for risk stratification [8]. In this exploratory analysis from the population-based STHLM3 study [13], we have addressed how well PSA, PSA density, and the novel Stockholm3 blood test can identify the subset of men with prostate cancer among those with at least moderate LUTS.

## 2 Patients and methods

We used data from the STHLM3 study [13]. STHLM3 is a prospective and population-based diagnostic study performed in Sweden during 2012–2015. A population-based sample of men aged 50–69 yr and without previous prostate cancer were invited, and 58 558 men participated. All participants with PSA ≥ 3 ng/mL were recommended to undergo prostate biopsies. The biopsy procedure included 10–12-core systematic biopsies taken from the peripheral zone of the prostate (apex, middle, and base). Biopsies were performed by experienced urologists, and specimens were analyzed by a single, senior, and highly experienced uropathologist (L.E.). Both urologists and the pathologist were blinded to PSA and Stockholm3 levels. Prebiopsy magnetic resonance imaging (MRI) was not included in the study protocol. Prostate volume was measured in milliliters using transrectal ultrasound.

As part of the Stockholm3 study, an International Prostate Symptom Score (IPSS) questionnaire was sent to all invitees. IPSS is a validated and extensively used self-reported symptom score calculated from the answers of seven questions regarding urinary symptoms and one regarding quality of life (Supplementary material). The urological symptoms evaluated are incomplete emptying, intermittency, frequency, urgency, weak stream, straining, and nocturia. The IPSS question on quality of life was disregarded in this study. Each question renders 0–5 points depending on symptom severity; thus, a total of 35 points can be obtained. The total score is categorized as low, medium, or high, and can then be used to grade LUTS as mild (IPSS 0–7 points), moderate (8–19 points), or severe (20–35 points). We previously reported on the distribution of IPSS scores in the study population [14].

PSA density was calculated as PSA (ng/mL) divided by prostate volume (ml). Stockholm3 is a commercially available blood test including clinical information (age, previous biopsy [1/0], family history [1/0], and prostate volume), protein levels (total PSA, free PSA, human Kallikrein2, MSMB, and MIC), and a polygenic score based on single nucleotide polymorphisms. The Stockholm3 test gives the risk in percent for the detection of ISUP grade ≥2 prostate cancer on systematic biopsies and has been validated externally [13], [15], [16]. We chose presented cutoffs a priori based on previous publications and clinical practice.

For this exploratory analysis, we included men with PSA ≥ 3 ng/mL, existing data on voiding symptoms (IPSS score), clinical data (age, previous biopsy history, prostate volume, and digital rectal examination), Stockholm3 score, and a biopsy report to study the associations between the levels of PSA, PSA density, and Stockholm3, with the risk of finding significant prostate cancer on biopsy. We defined significant prostate cancer as ISUP grade ≥2 and also report an alternative definition (ISUP grade ≥3).

Logistic regression was used to investigate the association of PSA, PSA density, and Stockholm3 with the risk of significant prostate cancer. Adjusted analyses included information on age (years) and previous biopsy (1/0) together with the predictor (PSA, PSA density, and Stockholm3). We calculated odds ratio for ISUP grade–specific cancer in relation to IPSS. Area under the receiver operating characteristic (ROC) curve (AUC) was used to assess discrimination for significant prostate cancer, and calibration was assessed visually. Differences in AUCs were assessed using the DeLong method. To determine the clinical value of the tests in this cohort, we used decision curve analysis.

STATA 14.0 were used as software for data management and statistical analysis.

## 3 Results

### 3.1 Demographics

Of the Stockholm3 participants, 77.5% (45 595/58 818) filled out the IPSS questionnaire. Among the 45 595 men who completed an IPSS form, we identified 4588 men with PSA ≥ 3 ng/mL who underwent a biopsy procedure. Among these men, we identified 1554 (36%) with at least moderate LUTS (IPSS ≥ 8), including 231 (5%) with severe LUTS (IPSS ≥ 20). The mean age was 64 yr (standard deviation 4.8), and 11% (*n* = 177) had undergone a previous prostate biopsy (Table 1).

**Table 1:** Clinical characteristics in 1554 men with moderate LUTS and PSA ≥ 3 ng/mL

Clinical characteristics				
Age (yr)	Mean, SD	64.2	,	4.8
PSA (ng/mL)	Median, IQR	4.2	,	2.3
PSA density (ng/mL^2^)	Median, IQR	0.09	,	0.06
Stockholm3, %	Median, IQR	0.10	,	0.1
Previous biopsy				
No	*n*, %	1373	,	88
Yes	*n*, %	177	,	11
Missing	*n*, %	4	,	0.3
IPSS	Median, IQR	12	,	8
Moderate LUTS (8–19)	*n*, %	1323	,	85
Severe LUTS (≥20)	*n*, %	231	,	15
Biopsy finding				
Benign	*n*, %	1045	,	67
ISUP grade 1	*n*, %	296	,	19
ISUP grade 2–3	*n*, %	173	,	11
ISUP grade ≥4	*n*, %	40	,	3

IPSS = International Prostate Symptom Score; IQR = interquartile range; ISUP = International Society of Urological Pathology; LUTS = lower urinary tract symptoms; PSA = prostate-specific antigen; SD = standard deviation.

### 3.2 Discrimination for prostate cancer

**Fig. 1:**
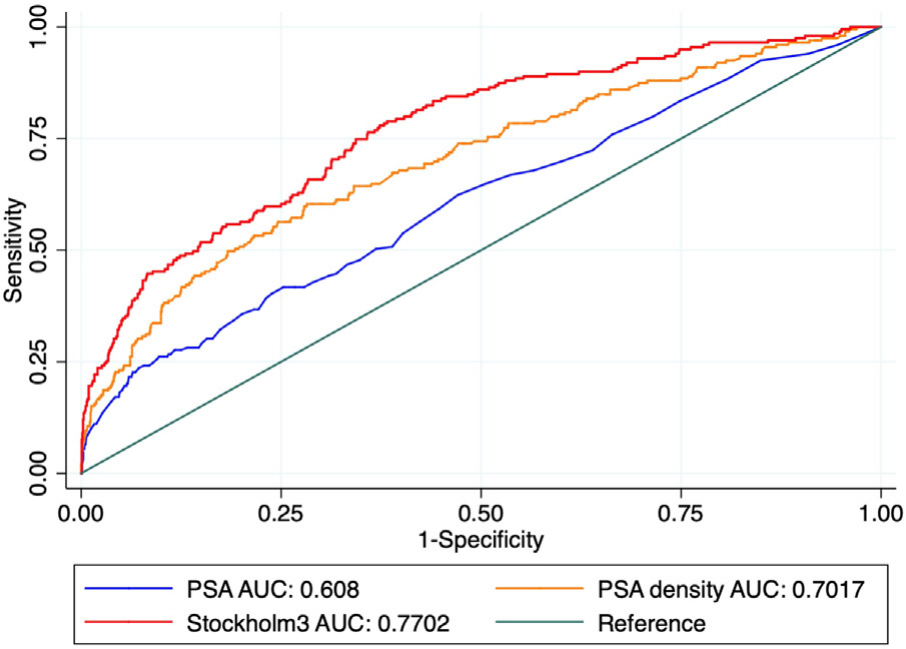
Discrimination for ISUP ≥ 2 cancer among 1554 men with PSA ≥ 3 ng/mL and at least moderate urinary tract symptoms (IPSS ≥ 8). AUC = area under the receiver operating characteristic curve; IPSS = International Prostate Symptom Score; ISUP = International Society of Urological Pathology; PSA = prostate-specific antigen.

The AUCs for discrimination of significant (ISUP grade ≥2) prostate cancer were 0.61 (95% confidence interval [CI] 0.56–0.65) for PSA, 0.70 (95% CI 0.66–0.74) for PSA density, and 0.77 (95% CI 0.73–0.80) for the Stockholm3 test. The differences in AUCs were all statistically significant for significant cancer of both definitions (pairwise comparisons; *p* < 0.02; Table 2). The corresponding ROC curves are shown in Fig. 1.

**Table 2:** Discrimination for significant prostate cancer comparing logistic regression models using total PSA, PSA density, and the Stockholm3 test

	ISUP grade group ≥2	ISUP grade group ≥3
	AUC (95% CI)	AUC (95% CI)
Total PSA	0.61 (0.56–0.65)	0.65 (0.58–0.72)
PSA density	0.70 (0.66–0.74)	0.72 (0.65–0.78)
Stockholm 3	0.77 (0.73–0.80)	0.77 (0.71–0.83)

AUC = area under the receiver operating characteristic curve; CI = confidence interval; ISUP = International Society of Urological Pathology; PSA = prostate-specific antigen.

In logistic regression analysis adjusted for age and previous biopsy, PSA, PSA density, and the Stockholm3 test showed independent values for predicting the presence of significant prostate cancer of both definitions (Supplementary Tables 1–3).

### 3.3 Clinical effects of using PSA, PSA density, or the Stockholm3 test for identifying men with LUTS at increased risk of prostate cancer

Clinical effects of using different PSA, PSA density, and Stockholm3 cutoffs to recommend prostate biopsy are illustrated in Table 3. The use of both PSA < 5 and PSA < 7 ng/mL for excluding men from prostate biopsy would result in missing more than half of detectable significant cancer (52% and 69%, respectively), saving 64–84% of performed biopsy procedures. The unbiopsied men would then have an 11% risk of finding significant prostate cancer.

**Table 3:** Detected cancers and individual risk of prostate cancer in 1554 men with PSA ≥ 3 ng/mL with LUTS by levels of PSA, PSA density, and Stockholm3 risk score

Groups by PSA, PSA density, and Stockholm3	No. of men, *n* (%)	ISUP 1	ISUP ≥ 2		ISUP ≥3	
		Nondetected cancers, *n* (%)	Nondetected cancers, *n* (%)	Individual risk in unbiopsied men (%)	Nondetected cancers, *n* (%)	Individual risk in unbiopsied men (%)
All men	1554 (100)	0/296 (0)	0/213 (0)	13.7	0/89 (0)	5.7
PSA (ng/mL) strata						
<5	996 (64)	196 (66)	110 (52)	11.0	39 (44)	3.9
<7	1298 (84)	252 (85)	148 (69)	11.4	55 (62)	4.2
PSA density (ng/mL^2^) strata						
<0.07	411 (26)	64 (22)	25 (12)	6.1	10 (11)	2.4
<0.1	879 (57)	157 (53)	67 (31)	7.6	25 (28)	2.8
<0.15	1308 (84)	243 (82)	125 (59)	9.6	44 (49)	3.4
Stockholm3 strata						
<0.11	820 (53)	123 (42)	42 (20)	5.1	17 (19)	2.1
<0.15	1002 (64)	158 (54)	74 (35)	7.4	26 (29)	2.6

ISUP = International Society of Urological Pathology; LUTS = lower urinary tract symptoms; PSA = prostate-specific antigen.

The use of a PSA density cutoff of 0.07 would spare 26% of men from undergoing a prostate biopsy, with a 6.1% risk of significant cancer among unbiopsied men. In a similar fashion, the use of the Stockholm3 test with a cutoff of 0.11 would result in sparing more than half (53%, *n* = 820) of men from biopsy, leaving unbiopsied men with a risk of finding significant cancer of 5.1% (Table 3).

On decision curve analysis, Stockholm3, PSA density, and PSA suggested a net benefit above the biopsy-all strategy at threshold probabilities of 5%, 7%, and 10%, respectively (Supplementary Fig. 1). Stockholm3 consistently showed higher net benefit than PSA density and PSA. These results suggest that Stockholm3 is better suited for guiding decision making about prostate biopsy than PSA density, which in turn is better than PSA.

### 3.4 Overdetection of low-grade prostate cancer

In order to decrease treatment-related harm and healthcare costs, it is of importance to minimize overdetection of low-grade prostate cancer. Using any risk-stratification tool in our dataset (PSA cutoff of 5 or 7 ng/mL, PSA density, or Stockholm3) decreases the detection of low-grade (ISUP grade 1) cancer. The degree of decreasing detection of low-grade cancer was roughly proportional to the degree of saved biopsies (Table 3). For example, the use of Stockholm3 with a cutoff of 0.11 would result in decreasing the detection of ISUP 1 tumors by 42% (*n* = 123 undetected ISUP grade 1 tumors).

### 3.5 Performance of the Stockholm3 test

**Fig. 2:**
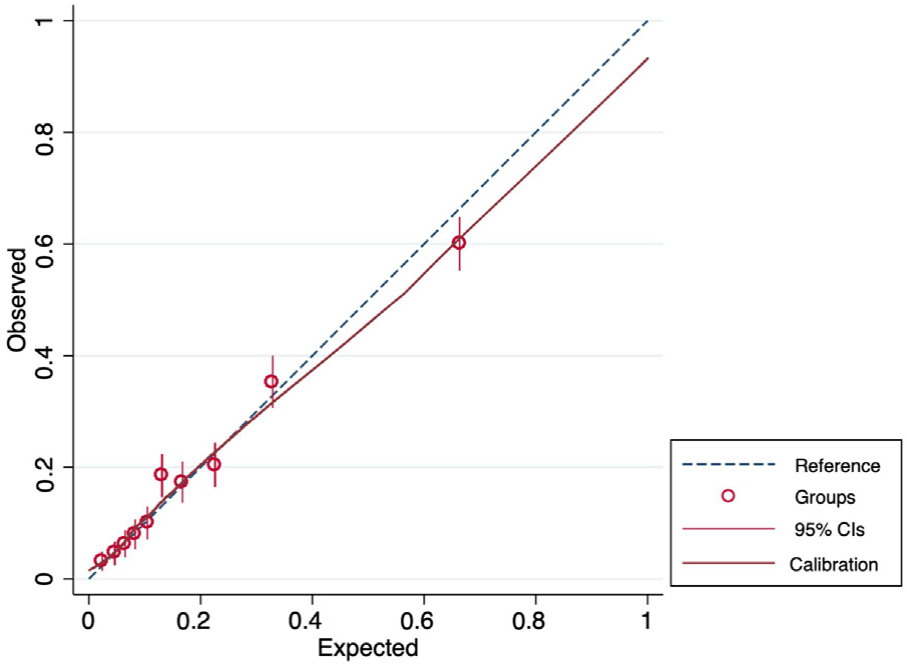
Calibration for ISUP ≥ 2 cancer detection on systematic biopsies among ≈1500 men with urinary tract symptoms (LUTS) by the Stockholm3 test. CI = confidence interval; ISUP = International Society of Urological Pathology; LUTS = lower urinary tract symptoms.

The AUC of the Stockholm3 test was 0.77 for the detection of significant prostate cancer of both definitions (Table 2 and Fig. 1). In this dataset, calibration was adequate with a slight tendency of underprediction (Fig. 2).

## 4 Discussion

In this exploratory analysis of a population-based screening trial, we report that the use of the Stockholm3 blood test or PSA density for the detection of significant prostate cancer among men with LUTS is superior to the use of PSA solely. The Stockholm3 test showed an independent value to predict the presence of significant prostate cancer, and had high discriminative properties (AUC 0.77) and adequate calibration. We further show that PSA density also outperforms PSA for identifying men with significant prostate cancer, showing good discrimination (AUC 0.71). Using PSA density or the Stockholm3 test instead of only PSA decreased the number of biopsy procedures performed and the number of low-grade cancers detected at the cost of delaying diagnosis of a few significant cancers.

Biopsying all men in this cohort would have led to *no* finding of significant prostate cancer in 86% of biopsied men. Moreover, 19% of the biopsied men received a diagnosis of low-grade prostate cancer. Especially in the light of the risk of overdetection and biopsy-related complications, this indicates a need for improved risk stratification.

As compared with performing prostate biopsies in all men with PSA ≥ 3 ng/mL, the use of Stockholm3 with a cutoff of 0.11 resulted in saving half of biopsy procedures at the cost of delaying diagnosis for a fifth of the significant cancers. The number of low-grade cancers decreased by 42% using this strategy, and the risk of significant cancer in men who did not undergo biopsy was 5%.

An alternative approach would be to use PSA density for risk stratification. In this case, the use of a PSA-density cutoff of ≥0.07 for performing biopsies would save a fourth of biopsies at the cost of delaying 12% of significant cancer diagnoses. The use of only a higher PSA cutoff (eg, PSA 5 ng/mL) for risk stratification resulted in delaying more than half of cancer diagnoses, with an individual cancer risk of 11% in unbiopsied men, thus being a less attractive alternative.

Current guidelines, for example, those from the European Association of Urology [8], often state that PSA should be recommended as a part of the workup of LUTS in well-informed men. While at least a fourth of men aged above 50 yr report moderate LUTS [14], such recommendations affect not only a large number of men, but also the detection of prostate cancer at large. In light of this, we argue that prostate cancer diagnostic strategies offered to men with LUTS should incorporate well-defined and well-performing diagnostic tools. In our study, both PSA density and the Stockholm3 test represent better alternatives than the use of PSA only for this objective.

Our study has some key strengths. First, it includes a population-based sample of men where everyone with PSA ≥ 3 ng/mL was invited for a systematic biopsy procedure, and the biopsy specimen was assessed by a single, highly experienced pathologist. Second, verification of LUTS was done using the most commonly used and best validated symptom score available. Third, the sample size was also enough to assess for a more conservative definition of significant cancer, lending robustness to the results. However, our study was not devoid of limitations. First, the true prevalence of significant prostate cancer is unknown, and the definition of significant cancer is frequently debated. We try to assess this by including an alternative definition of significant cancer. However, we acknowledge the lack of data on disease progression in our study. Second, we use traditional (systematic) biopsies for disease verification despite the rapid development of MRI-targeted biopsy approaches. However, we argue that the definition of significant cancer might be even harder when using imaging-based strategies, while the evidence base for such strategies is still evolving. Further, we find no obvious reason to believe that the comparative performance between PSA, PSA density, and the Stockholm3 test is affected by the biopsy technique.

## 5 Conclusions

We find that PSA performs poorly for identifying significant prostate cancer in men with LUTS. The use of PSA density or the Stockholm3 blood test improves the detection of significant cancer as compared with using PSA only. Guideline bodies that today recommend PSA testing in men with LUTS might consider including improved risk stratification in order to enhance counseling of men.

***Author contributions***: Tobias Nordström had full access to all the data in the study and takes responsibility for the integrity of the data and the accuracy of the data analysis.

  *Study concept and design*: Nordström, Engel, Eklund, Grönberg.

*Acquisition of data*: Nordström, Egevad, Palsdottir, Grönberg.

*Analysis and interpretation of data*: Nordström, Palsdottir, Eklund, Engel, Bergman.

*Drafting of the manuscript*: Nordström, Engel, Bergman, Eklund, Grönberg.

*Critical revision of the manuscript for important intellectual content:* Nordström, Engel, Bergman, Egevad, Eklund, Palsdottir, Grönberg.

*Statistical analysis*: Nordström, Palsdottir.

*Obtaining funding*: Nordström, Grönberg, Eklund.

*Administrative, technical, or material support:* Grönberg, Eklund, Nordström.

*Supervision*: Nordström, Grönberg.

*Other*: None.

  ***Financial disclosures***: Tobias Nordström certifies that all conflicts of interest, including specific financial interests and relationships and affiliations relevant to the subject matter or materials discussed in the manuscript (eg, employment/affiliation, grants or funding, consultancies, honoraria, stock ownership or options, expert testimony, royalties, or patents filed, received, or pending), are the following: Henrik Grönberg, Martin Eklund, and Tobias Nordström own shares in A3P Biomedical. A3P Biomedical develops and sells tools for improved prostate cancer care.

  ***Funding/Support and role of the sponsor:*** This study was supported by grants from the Strategic Research Programme on Cancer (StratCan), Karolinska Institutet; the Linné Centre for Breast and Prostate Cancer (CRISP, 70867901), Karolinska Institutet; the Swedish Research Council (K2010-70X-20430-04-3; 2015-03292); the Swedish Cancer Society (11-0287; 2015/649); Stiftelsen Johanna Hagstrand och Sigfrid Linners Minne; and FORTE 2015-00184. The funding source had no role in the study design; collection, analysis, or interpretation of data; writing of the report; or the decision to submit the article for publication. The researchers were all independent from the funding source.

  ***Acknowledgments:*** We thank all the study participants; the STHLM3 core management group for taking care of all contacts with participants, organizing the databases, and performing analyses; KI Biobank at Karolinska Institutet for taking care of blood sampling and sample handling; Karolinska University Hospital Laboratory for organizing sample handling and analysis; the STHLM3 outpatient urologists for taking care of patients and performing biopsies; and Unilabs AB for biopsy handling.

## CRediT authorship contribution statement

**Tobias Nordström:** Conceptualization, Methodology, Software, Formal analysis, Investigation, Resources, Data curation, Writing - original draft, Writing - review & editing, Visualization, Supervision, Project administration, Funding acquisition. **Jan Chandra Engel:** Conceptualization, Writing - original draft, Writing - review & editing. **Martin Bergman:** Writing - original draft, Writing - review & editing, Visualization. **Lars Egevad:** Investigation, Resources, Writing - review & editing. **Markus Aly:** Writing - review & editing. **Martin Eklund:** Conceptualization, Methodology, Software, Investigation, Resources, Data curation, Writing - review & editing, Supervision, Project administration, Funding acquisition. **Thorgerdur Palsdottir:** Methodology, Software, Validation, Software, Formal analysis, Investigation, Data curation, Writing - review & editing, Visualization. **Henrik Grönberg:** Conceptualization, Investigation, Resources, Writing - review & editing, Supervision, Project administration, Funding acquisition.
