# Supplementary material for: Identifying Prostate Cancer Among Men with Lower Urinary Tract Symptoms
Source: Eur Urol Open Sci. 2021 Jan 1;24:11–6. doi: 10.1016/j.euros.2020.12.004 (PMC8317798; doi:10.1016/j.euros.2020.12.004)

Complete logistic regression

Supplementary Table 1: Logistic regression models on risk of significant prostate cancer adjusted for

age, previous biopsy status, prostate volume and PSA (ng/ml)

|  | ISUP grade group ≥2 | | | ISUP Grade group ≥3 | | |
| --- | --- | --- | --- | --- | --- | --- |
|  | OR | 95% CI | | OR | 95% CI | |
| Age | 0.99 | 0.97 | 1.03 | 0.99 | 0.96 | 1.05 |
| Previous biopsy status | 0.19 | 0.08 | 0.43 | 0.17 | 0.04 | 0.71 |
| PSA, ng/ml | 1.1 | 1.07 | 1.15 | 1.03 | 1.01 | 1.05 |

Supplementary Table 2: Logistic regression models on risk of significant prostate cancer adjusted for

age, previous biopsy status, prostate volume and PSA density (ng/ml^2^)

|  | ISUP grade group ≥2 | | | ISUP Grade group ≥3 | | |
| --- | --- | --- | --- | --- | --- | --- |
|  | OR | 95% CI | | OR | 95% CI | |
| Age | 1.01 | 0.98 | 1.05 | 1.01 | 0.96 | 1.05 |
| Previous biopsy status | 0.20 | 0.08 | 0.46 | 0.35 | 0.12 | 0.96 |
| PSA density, ng/ml^2^ | 1.07 | 1.05 | 1.09 | 1.05 | 1.03 | 1.06 |

Supplementary Table 3: Logistic regression models on risk of significant prostate cancer adjusted for

age, previous biopsy status, prostate volume and Stockholm3 (%)

|  | ISUP grade group ≥2 | | | ISUP Grade group ≥3 | | |
| --- | --- | --- | --- | --- | --- | --- |
|  | OR | 95% CI | | OR | 95% CI | |
| Age | 0.98 | 0.95 | 1.02 | 0.98 | 0.93 | 1.03 |
| Previous biopsy status | 0.35 | 0.14 | 0.88 | 0.43 | 0.10 | 1.78 |
| Stockholm3 (%) | 1.05 | 1.04 | 1.06 | 1.05 | 1.04 | 1.06 |

**Supplementary Figure 1:**

Decision curve analysis for the Stockholm3 test (red), PSA density (orange) and PSA (blue) when predicting clinically significant prostate cancer. As a comparison, the net benefits from a biopsy-all strategy (solid orange line) and a biopsy-none strategy (solid green line) are shown. Clinically significant cancer was defined as Gleason Score ≥7. PSA – prostate-specific antigen.

(A)


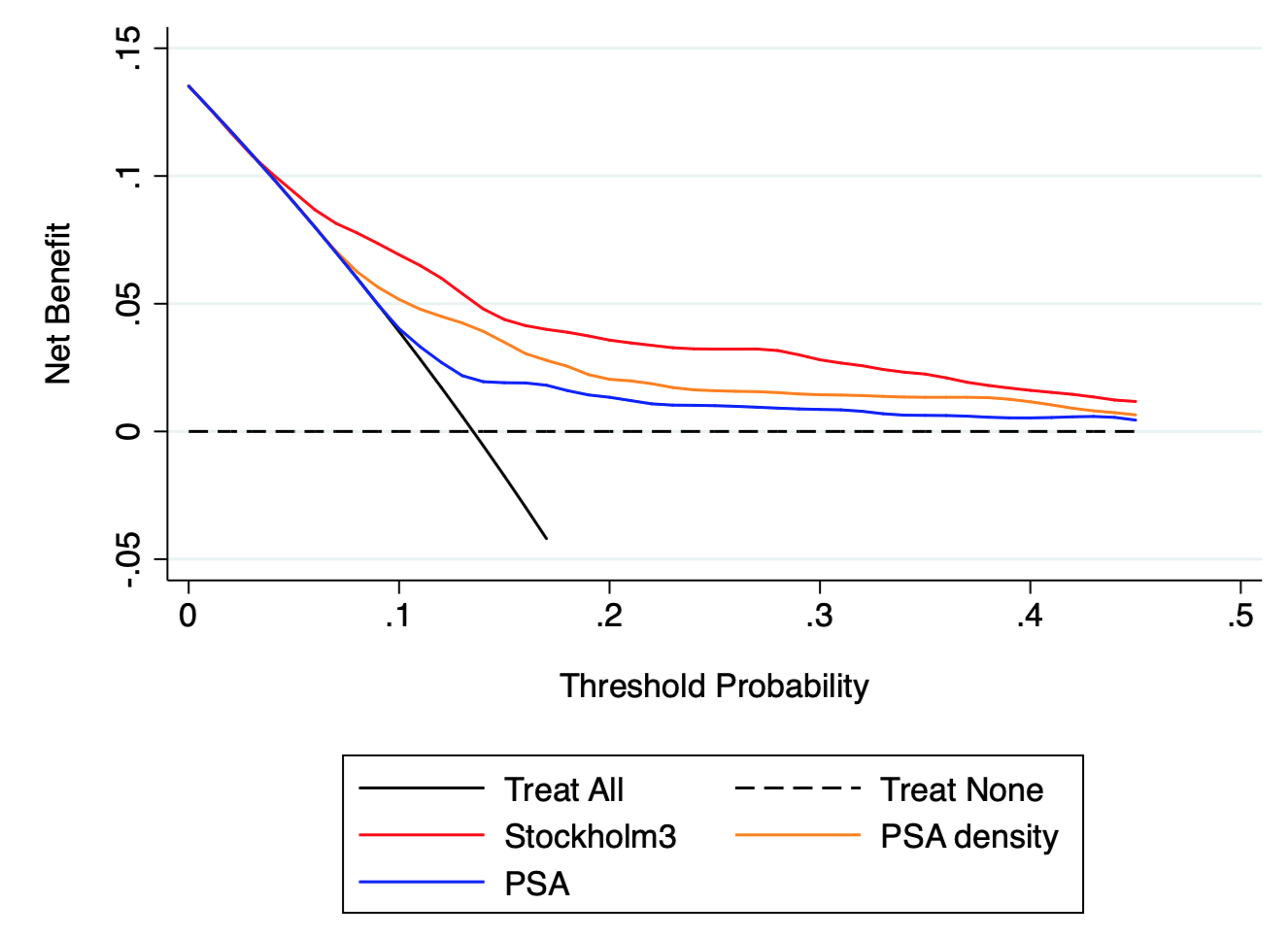

Supplement: Supplementary file 1 [file mmc1.docx]
